# Docking Analysis and Multidimensional Hybrid QSAR Model of 1,4-Benzodiazepine-2,5-Diones as HDM2 Antagonists

**Published:** 2012

**Authors:** Yujie Dai, Nan Chen, Qiang Wang, Heng Zheng, Xiuli Zhang, Shiru Jia, Lilong Dong, Dacheng Feng

**Affiliations:** a *Key Laboratory of Industrial Fermentation Microbiology (Tianjin University of Science and Technology), Ministry of Education, College of Bioengineering, Tianjin University of Science and Technology, Tianjin 300457, P.R. China.*; b *School of Life Science and Technology, China Pharmaceutical University, Nanjing 210009, P.R. China.*; c *Department of Biochemistry, University of Missouri-Columbia, Columbia, MO 65211, USA.*; d *School of Pharmaceutical Sciences, Hebei Medical University, Shijiazhuang 050017, P.R. China.*; e *College of Chemistry and Chemical Engineering, Shandong University, Jinan 250100, P.R. China. *

**Keywords:** p53-HDM2 interaction, Docking, QSAR, 1, 4-benzodiazepine-2, 5-diones, CDOCKER

## Abstract

The inhibitors of p53-HDM2 interaction are attractive molecules for the treatment of wild-type p53 tumors. In order to search more potent HDM2 inhibitors, docking operation with CDOCKER protocol in Discovery Studio 2.1 (DS2.1) and multidimensional hybrid quantitative structure-activity relationship (QSAR) studies through the physiochemical properties obtained from DS2.1 and E-Dragon 1.0 as descriptors, have been performed on 59 1,4-benzodiazepine- 2,5-diones which have p53-HDM2 interaction inhibitory activities. The docking results indicate that π-π interaction between the imidazole group in HIS96 and the aryl ring at 4-N of 1,4-benzodiazepine-2,5-dione may be one of the key factors for the combination of ligands with HDM2. Two QSAR models were obtained using genetic function approximation (GFA) and genetic partial least squares (G/PLS) based on the descriptors obtained from DS2.1 and E-dragon 1.0, respectively. The best model can explain 85.5% of the variance (R ^2^_adj_ ) while it could predict 81.7% of the variance (R^ 2^_cv_ ). With this model, the bioactivities of some new compounds were predicted.

## Introduction

The p53 is a multifunctional protein that regulates genes which can induce either cell cycle arrest or apoptosis (1-3). In human cells, p53 activity is normally modulated by the human double minute-2 (HDM2) protein, the homologue of murine double minute-2 (MDM2) protein (4). HDM2 binds to and blocks the p53 transactivation domain, inhibiting its transcriptional activity. The dissociation of the binding between these two proteins is therefore an attractive therapeutic target for the treatment of wild-type p53 tumors (5). This action can be implemented through neutralizing antibodies (6), p53 peptides (7) or protein fusions (8, 9), which leads to the control of proliferation. Additionally, antisense HDM2 oligonucleotides increase both p53 levels and activity by reducing HDM2 protein levels (10, 11). Since small molecular antagonists of HDM2 are easily synthesized, some investigations on them have been reported, but many of these molecules have low potency and limited cellular activity (12-14). Most recently, some reports describing a series of 1,4-benzodiazepine-2,5-dione compounds (BDPs) that act as the potent antagonists of the HDM2–p53 interaction (15). A library of BDPs was designed by some investigators using directed diversity method (16-18). These compounds were synthesized and screened with thermofluor screening technology (19) and further evaluated with a fluorescence polarization (*FP*) assay. In order to further understand the antagonizing mechanism of these compounds with HDM2-p53 interaction, Grasberger (11) *et al. *studied the crystal structure of BDPs with HDM2. The crystal structure confirmed that the BDPs occupy the p53 peptide binding site and they can mimic the *α*-helix of p53 peptide and may represent a promising scaffold to develop HDM2 antagonists. The three substituted aryl rings of the BDP overlay very closely with PHE19, TRP23 and LEU26 in the p53 binding pocket of HDM2 (20) and some structure-activity relationship (SAR) studies were deduced and explained (15). Recently, Wang *et al. *(21) conducted a benzodiazepinedione/peptide-based 3D-QSAR analysis using the comparative molecular field analysis (CoMFA) and comparative molecular similarity index analysis (CoMSIA), however, their molecular poses of ligands in the pocket of HDM2 from docking and crystal structure were only considered a little by taking a receptor-guided consensus dynamics alignment since the poses of the alignments method are different from those of docking results. On the other hand, there is no explicit quantitative equation reported for the QSAR of BDPs as inhibitors of the p53-HDM2 although some quantitative structure-activity relation (QSAR) models have been reported for other HDM2 inhibitors (22, 23). Whereas, BDPs are a kind of most potent HDM2 inhibitors, a high quality equation as QSAR model is necessary for finding new BDPs as potent HDM2 inhibitors. It is also important for new HDM2 inhibitors from other structural compounds.

Lately, with the development of computer hardware and computational theory, many molecule structural properties can be obtained through the computational process. Some software products provide these functions, such as Discovery studio (24), MOE (25), SYBYL (26) and E-Dragon (27), *etc*. For example, Discovery Studio 2.1 can calculate more than 100 kinds of molecular properties and more than 1600 molecular properties can be obtained from E-Dragon 1.0. These properties can be used expediently as descriptors to construct a QSAR model for drug screening. In 2009, Roys (28) calculated 2D and 3D properties of 116 diverse classes of aromatase inhibitors. With the assistance of genetic function approximation (GFA) and genetic partial least squares (G/PLS), QSAR models for non-steroid compounds were constructed using these 2D and 3D descriptors. The best (externally) predictive model was a GFA model with spline option using combined set (2D and 3D) descriptors and its predictive *R*^2^ (*R*_pred_^2^) reached 0.687. Dai *et al*. (29) used the computed molecular properties and docking scores (CDOCKER interaction energy) constructed a 2D-3D hybrid QSAR model for steroid aromatase inhibitors with the correlation coefficient of *R*^2^ = 0.729 and Leave-one-out Cross-Validation of *R*^2^ = 0.667. In these two studies, GFA was successfully employed to select the best combined set of descriptors from large number of physiochemical properties for the construction of high quality QSAR models. In order to construct a quantitative predictive model for the bioactivity of new 1,4-benzodiazepine-2,5-dione compounds as HDM2 antagonists, two QSAR models were built in this paper based on the computational properties of 59 1,4-benzodiazepine-2,5-dione compounds with known bioactivities. In addition, the molecular docking of BDPs to the HDM2 cleft was conducted using CDOCKER docking protocol in Discovery Studio 2.1(DS2.1) and the interaction of these compounds was analyzed with LigPlot (30).

## Experimental

*Dataset and descriptors*

The 1,4-benzodiazepine-2,5-dione chiral ligands with known inhibitory activities upon HDM2-p53 interaction reported in the literature (4, 15, 31, 32) were used as the model dataset for the present study (Table 1). The experimental protocol for the determination of inhibitory activities for all the compounds was the *FP *assay. The inhibitory values in *IC*_50_ (μM) were converted to the logarithmic scale *pIC*_50_ (mM) before being used for the subsequent QSAR analyses as the response variable. Various physiochemical properties of the ligands obtained using the protocol of Calculate Molecular Properties in DS2.1 and E-Dragon 1.0 respectively, were selected as candidate descriptors used for QSAR construction. These physiochemical properties include 1D (Element counts, functional group counts) 2D (*AlogP, LogD, Num_StereoAtoms, Num_Rings, Molecular_Weight, Molecular_SurfaceArea, Num_H_Acceptors, Num_H_Donors, Num_RotatableBonds, *2D autocorrelations, *topological descriptors such as BIC, V_ADJ_equ, V_DIST_equ, CHI_3_P, CHI_V_3_P, CHI_1, CHI_3_C, CIC, IAC_Mean, IAC_Total, IC and SIC, etc*.) and 3D (3D-NoRSE descriptors, *Dipole, Jurs descriptors, shadow indices and Molecular_Volume, etc.*) parameters. All the definition of these descriptors can be seen in the Help of DS2.1 and E-Dragon 1.0 software. For the calculation of 3D descriptors, multiple conformations of each molecule were generated using the Generate Conformations protocol with optimal search as a conformational search method. Each conformer was subjected to an energy minimization procedure using smart minimizer under the consistent force field (CFF) to generate the lowest energy conformation for each structure. The charges were calculated according to the Gasteiger method.

*Docking process of 1,4-benzodiazepine-2,5-diones to HDM2 protein*

**Figure 1:**
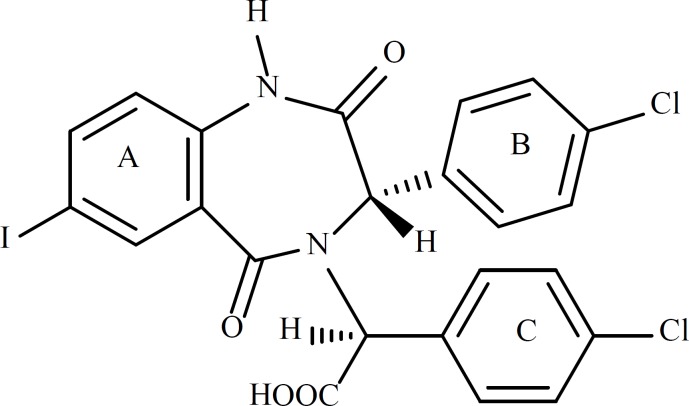
Structure of Ligand 25

The crystal structure of HDM2 in complex with a benzodiazepine ligand ((S)-2-(4-chlorophenyl)-2-((S)-3-(4-chlorophenyl)-7-iodo-2,5-dioxo-2,3-dihydro-1H-benzo[e][1,4]diazepin-4(5H)-yl) acetic acid, ligand 25 in Table 1, Figure 1) has been obtained from the RCSB protein data bank (http://www.pdb.org, ID:1T4E). 

The docking studies were conducted using CDOCKER of Receptor-ligand Interactions protocol in DS 2.1(33). The ligands and the HDM2 protein were all pretreated initially. For ligand preparation, the 3D structures of all the 1,4-benzodiazepine-2,5-diones were generated with ChemBioOffice 2008 (34) and optimized with AM1 method. Thereafter, the ligands were treated with Prepare Ligands protocol in DS 2.1. All the duplicate structures were removed and the options for ionization change, tautomer generation, isomer generation and 3D generator have been set true. For protein preparation, the hydrogen atoms were added with the pH in the range of 6.5-8.5 with DS2.1. HDM2 protein was defined as a total receptor and the site sphere was built with diameter of 10 Å based on the ligand 25. Then, the preexisting ligand 25 was removed. From the receptor-ligand interaction section of DS 2.1, CDOCKER was chosen and all the default operating parameters were used unless pre-declared. During the docking process, a freshly prepared ligand (compound from the dataset in Table 1) prepared by us was placed. CHARMm was selected as the force field. The molecular docking was performed with a simulated annealing method to minimize the CDOCKER energy (*E*_CD_) for obtaining an optimum pose. This method was independently described by Scott Kirkpatrick *et al. *in 1983 (35) and by Vlado Cerny in 1985 (36). The method is a technique suitable for the optimization problems of large scale, especially ones where a desired global extremum is hidden among many, poorer, local extrema. The name comes from annealing in metallurgy, a procedure involving heating and controlled cooling of a material to increase the crystal orders and reduce their defects. The heat makes the atoms to become unstuck from their initial positions with a local minimum of internal energy and move randomly through states of higher energy. The slow cooling procedure gives them ample time for redistribution of the atoms to find configurations with lower internal energy than the initial one. Similar to this physical process, each step of the simulated annealing algorithm changes the current solution through a random “nearby” solution with a probability that relies on both the difference between the corresponding function values and a global parameter *T *(named as temperature), which is gradually decreased during the process. The changes of current solution depend on *T *and are almost random when *T *is large, but increasingly “downhill” (for a minimization value) as *T *comes to zero. The chance for “uphill” moves potentially and prevents the method from becoming stuck at a local optimal value. In this study, the heating steps were set as 2000 with 700, heating target temperature. The cooling steps were set as 5000 with 300 as the cooling target temperature. Ten molecular docking poses saved for each ligand were ranked according to -CDOCKER energy (-*E*_CD_). The pose with the highest -*E*_CD_ was chosen as the most suitable pose for the subsequent pose analysis. After the end of molecular docking, the interactions of the docked receptor (HDM2) with ligand were analyzed with LigPlot. 

*QSAR model development*

For a comparatively small quantity of samples with large available variables, the number of variables (descriptors) should be reduced far smaller than that of samples so that the obtained equation has the statistical meaning. But it is difficult to choose which property is more suitable as the descriptor to build QSAR models. Recently, this problem can be solved with genetic function approximation (GFA) technique. The principles about GFA can be seen elsewhere (37, 38). It involves the multivariate adaptive regression algorithm combining with the genetic algorithm (GA) to evolve the population of equations (each containing only a subset of variables) that best fit the training set data. With this method, a series of potential solutions to a problem (the population of organisms) are derived and tested repeatedly until an approximate optimal solution is found. In this study, 59 1,4-benzodiazepine-2,5-dione chiral compounds in Table 1 were selected as the training set. There are more than 120 2D and 3D physiochemical properties obtained from the protocol of Calculate Molecular Properties in DS 2.1 and 1621 physiochemical properties obtained from E-Dragon 1.0. But only a subset of these properties is statistically significant according to the correlation with the compounds activities. Some redundant parameters from the protocol of Calculate Molecular Properties in DS 2.1 were omitted using GFA protocol in DS 2.1 in order to save time and space and to decrease the complexity of the QSAR model (The reduction of 1D-3D physiochemical descriptors from E-Dragon 1.0 to build the QSAR model was conducted using the Material Studio 4.0 (39)). 

*Statistical quality evaluation and model validation*

For a successful QSAR model, it should be robust enough to be capable of making accurate and reliable predictions of the biological activities, thus, the predictive capacity of the developed QSAR models from the training set should be validated. There are several methods to confirm the quality of QSAR model. In our experiments, Friedman lack-of-fit (*LOF*) was used for the selection of GFA-derived equations, while for G/PLS equations, the correlation coefficient *R*2 and the adjusted *R*^2^^(^R ^2^_adj_ ), were taken as objective functions to select an equation. Leave-one-out cross-validation *R*^2^ (R ^2^_cv_ ) was employed to validate the predictivity of generated QSAR model equations. The *LOF *[40] is designed to resist over-fitting, which is a problem often encountered in constructing statistical models. Since the number of descriptors available in HDM2 inhibitor QSAR analysis normally exceeds the number of observations (training set compounds), the ability to prevent over-fitting of GFA is critical to the successful construction of a statistically significant QSAR model. The smoothing factor was set to 0.5, which controls the model size, and GFA was used to optimize the QSAR models having different numbers of descriptor terms. For a given smoothing factor, the optimization of a QSAR model was considered to be realized when descriptor usage became constant and independent of an increasing number of crossover operations. All the descriptors in the QSAR trial descriptor pool were used as linear terms during the GFA to generate QSAR models.

## Results and Discussion

*Analysis of the binding site*

**Figure 2:**
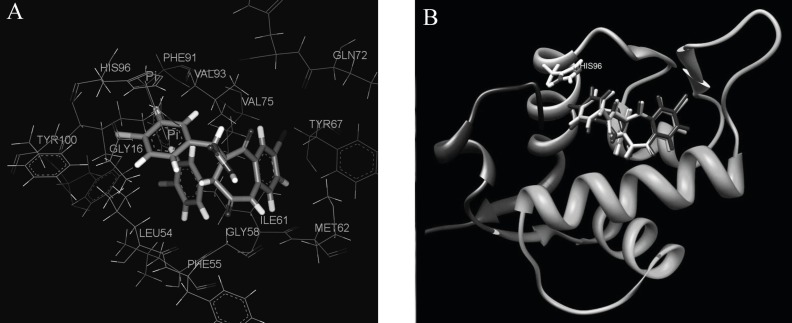
HDM2 binding model of Ligand 25 (25 is displayed in stick. H, white; C, gray; O, red; N, blue; Cl, green; iodo, and purple; PDB ID: 1T4E). (A) The specific residues surrounding 25 within 4Å (HIS96 is displayed in stick. π-π interaction is displayed in orange line and H-bond is in green dash line); (B) Comparison of Ligand 25 poses from X-ray crystal structure (red) and from CDOCKER docking (gray) and HIS96 is also shown in stick as location reference (The image was made with Chimera 1.3(41)).

To understand the molecular details of HDM2 binding through the BDPs, the binding site view was made using the DS visualizer 2.5 (Figure 2).

**Figure 3:**
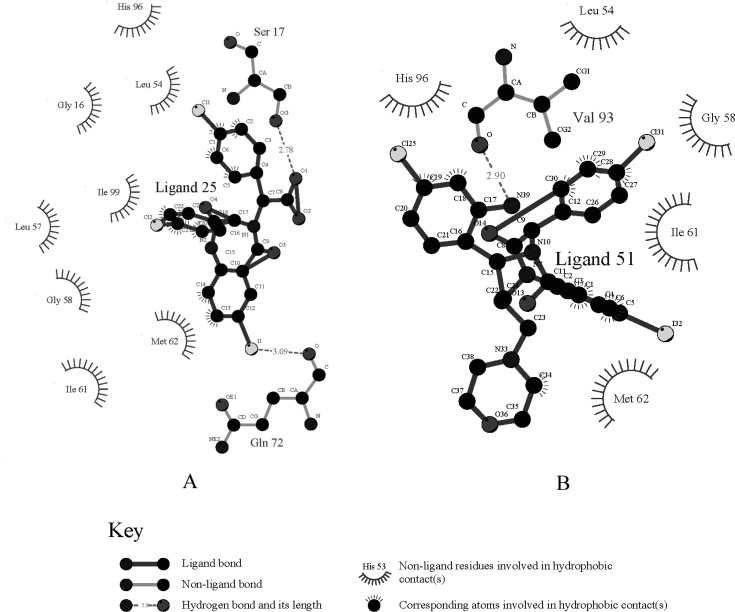
The interaction of the ligands with HDM2 protein (The image was made with LigPlot (30). A: ligand 25, B: ligand 51).

 The specific cleft to which the ligand binds (within 4 Å), contains both polar (GLY16, SER17, GLY58, ILE61, TYR67, GLN72, HIS73, HIS96, ILE99, TYR100) and nonpolar (LEU54, PHE55, MET62, VAL75, VAL93, PHE86, PHE97) amino acids and the inhibitor (contains phenyls A, B and C) occupies the same pockets as the peptide side-chains PHE 19, TRP 23, and LEU 26 of p53 (Figure 2A) ( 20). The HDM2 interaction with the ligand 25 was analyzed with LigPlot and it was found that the nonspecific hydrophobic contacts are largely responsible for their interaction. The hydrophobic contacts of HIS 96, LEU 54, GLY 16 with the ring of phenyl C, ILE 99, Leu 57, GLY 58 with phenyl B, and ILE 61, MET 62 with phenyl A are shown in Figure 3A. There are also two hydrogen bonds between the carboxyl group of Ligand 25 and hydroxyl group of SER17, iodine of Ligand 25 and hydroxyl group of GLN 72 respectively. It can be concluded that the binding cleft of HDM2 is predominantly hydrophobic and largely nonspecific Van Der Waals contacts are responsible for the interaction between the ligand and HDM2 hydrophobic pocket (11).

The 1,4-benzodiazepine-2,5-dione compounds (BDPs) used to construct QSAR models are shown in Table 1. All the ligands listed in Table 1 are different from each other in R1 to R4, which lead to the different conformations adopted in the cleft of HDM2, therefore, the interactions with HDM2 are different and the bioactivities are varied.

**Table 1:** Structural features of the 1,4-benzodiazepine-2,5-diones (4, 15, 27, 28) having HDM2 inhibitory activity

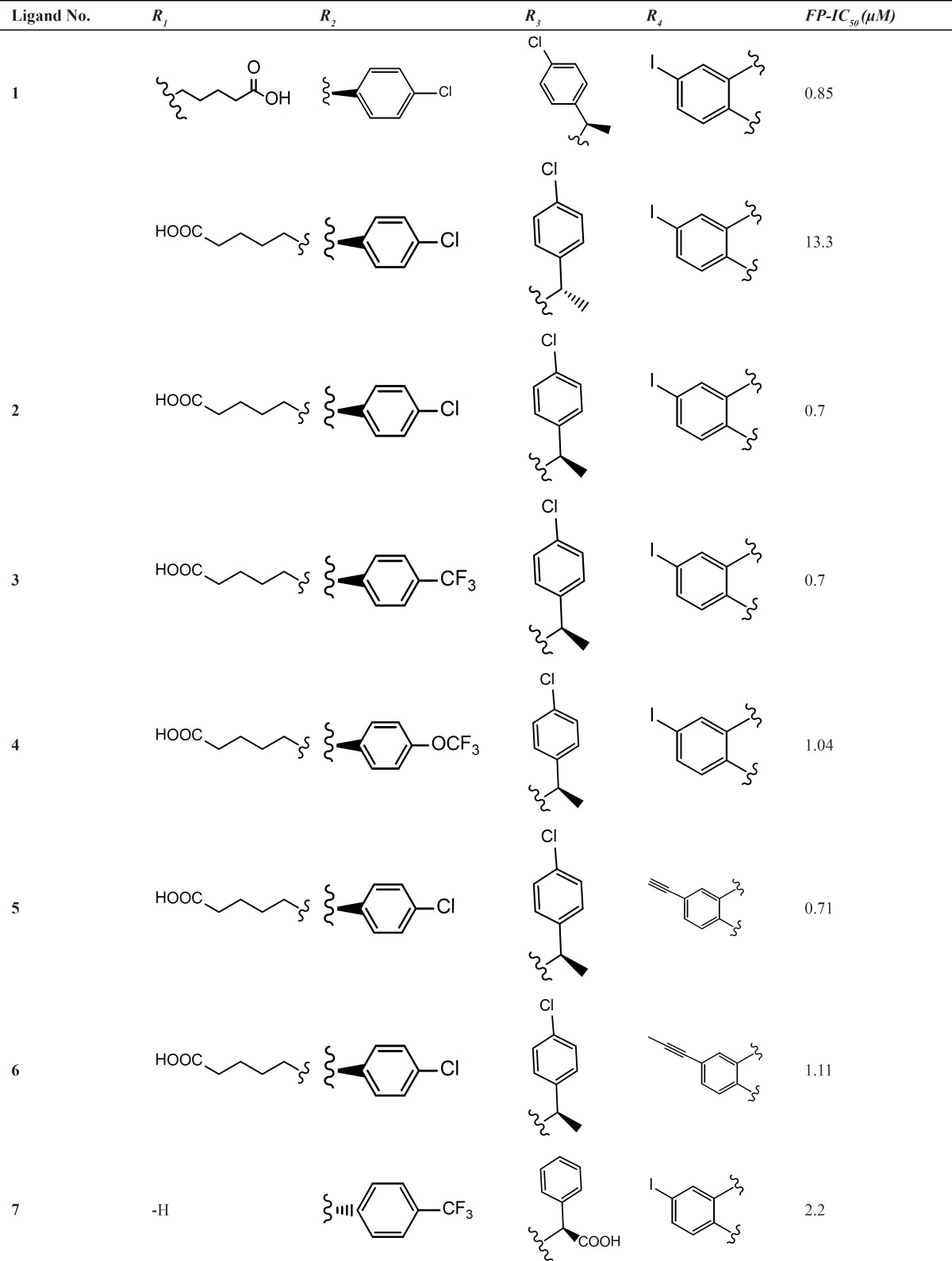

*Analysis of the optimum molecular docking poses of different ligands*

The CDOCKER algorithm was applied to dock the ligands listed in Table 1. This docking technique needs a site sphere surrounding the ligand (the radius was set to 10 Å in this study) and this technique was tested with the ligand 25. Our docking pose of freshly prepared model of ligand 25 with CDOCKER also corroborates with that in crystal structure (*RMSD *= 0.425) indicating the reliability of this docking procedure (Figure 2B). 

Each ligand in Table 1 was docked as described previously and the pose with the highest -*E*_CD_ was considered as the optimum pose for each ligand. For the ligands in the top bioactivity range, such as ligands 25, 51 and 55 (*IC*_50_ < 0.5 μM), there exists π-π interaction between the imidazole group in HIS96 and the aryl ring in R_3_ (the occasion for ligand 25 is seen in Figure 2A). It can be considered as one of the key factors for the combination of the ligands with HDM2. Although the pocket of HDM2 is mainly hydrophobic, the additional hydrogen bonds can be formed. By introducing an amine functional group in the ortho position of aryl ring in *R*_3_, an additional hydrogen bond with VAL93 is formed for ligand 51 or 55, which is consistent with the previous prediction in literature (Figure 3B) (33).The extra hydrogen bond formation increased the inhibitory potency of these ligands.

**Figure 4:**
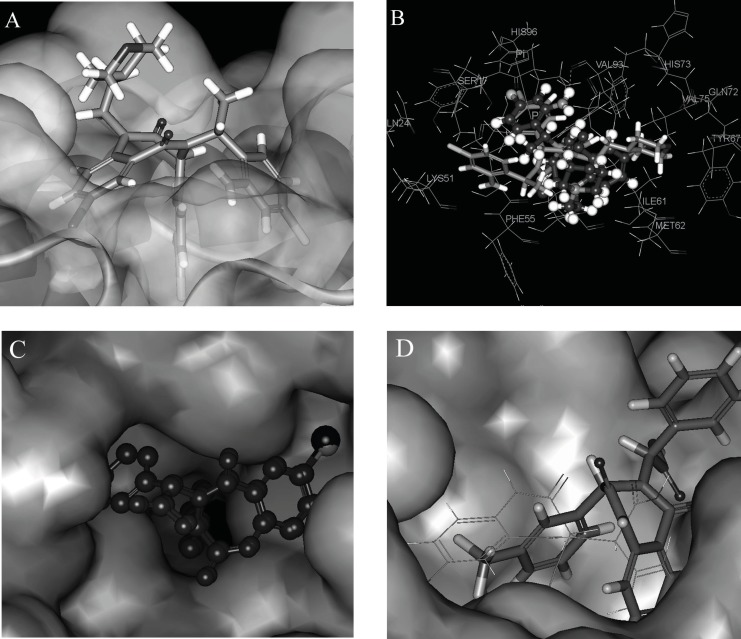
Poses of different ligands in the active site of HDM2. (A) Surface representation of Hdm-ligand 51 complex the protein (the morpholine group of ligand 51 is out of the HDM2 cleft). (B) Docking pose difference between ligands 51 (violet) and 52 (grey). Instead of the aryl ring in *R*_3_ of 51, the aryl ring in R_2_ formed the π-π interaction with the imidazole group of HIS96 in ligand 52. (C) Comparison of docking poses of ligand 25 (red, I in purple) with 35 (blue, Cl in green). (D) docking poses comparison of ligand 12 (in line) with ligand 15 (in stick).

The *R*_1_ changes in fatty acid, alkyl ether, alkyl (acyl) morpholine, alkyl (acyl) piperazine *etc. *(ligands 48-59). They are introduced in the ligand merely for improving the water-solubility of the ligands (33). From the docking position, we can see that these groups have nearly little contact with the acceptor protein (Figure 4A shows the morpholine group in ligand 51 stretches out of the HDM2 cleft), and in turn, have little effect on enhancing the ligand affinity. On the other hand, they improve the solvent effect, which has the negative effect on the ligands’ affinity to the target.

The chirality change at *R*_2_ and *α*-C of *R*_3_ also alters the bioactivity seriously, for example, ligands 49 and 50, 51 and 52, 53 and 54, are different from each other in *R*_2_ or *α*-C of *R*_3 _correspondingly (Figure 4B shows the docking pose difference between the ligands 51 and 52). Instead of the aryl ring in *R*_3_ of 51 (in violet), the aryl ring in *R*_2_ formed the π-π interaction with the imidazole group of HIS96 in ligand 52 (grey). This conformation makes the extra hydrogen bond existed in ligands 51 which can’t be formed for ligand 52 that may lower the affinity of the ligand with HDM2.

Figure 4C shows the comparison of docking poses of I substituted (ligand 25) and Cl substituted (ligand 35) phenyl A ligands. From the comparison of the docking results, it was found that iodine group in phenyl A is suitable for the cleft space. When the iodine group of phenyl A was replaced by other halogen, cyanogen, amide, alkyl or alkyne groups, the ligand sizes could not match the HDM2 cleft as exactly as iodine, therefore, the interaction between the ligand and HDM2 was decreased and as a result, the HDM2 inhibitory activities were lowered.

**Figure 5:**
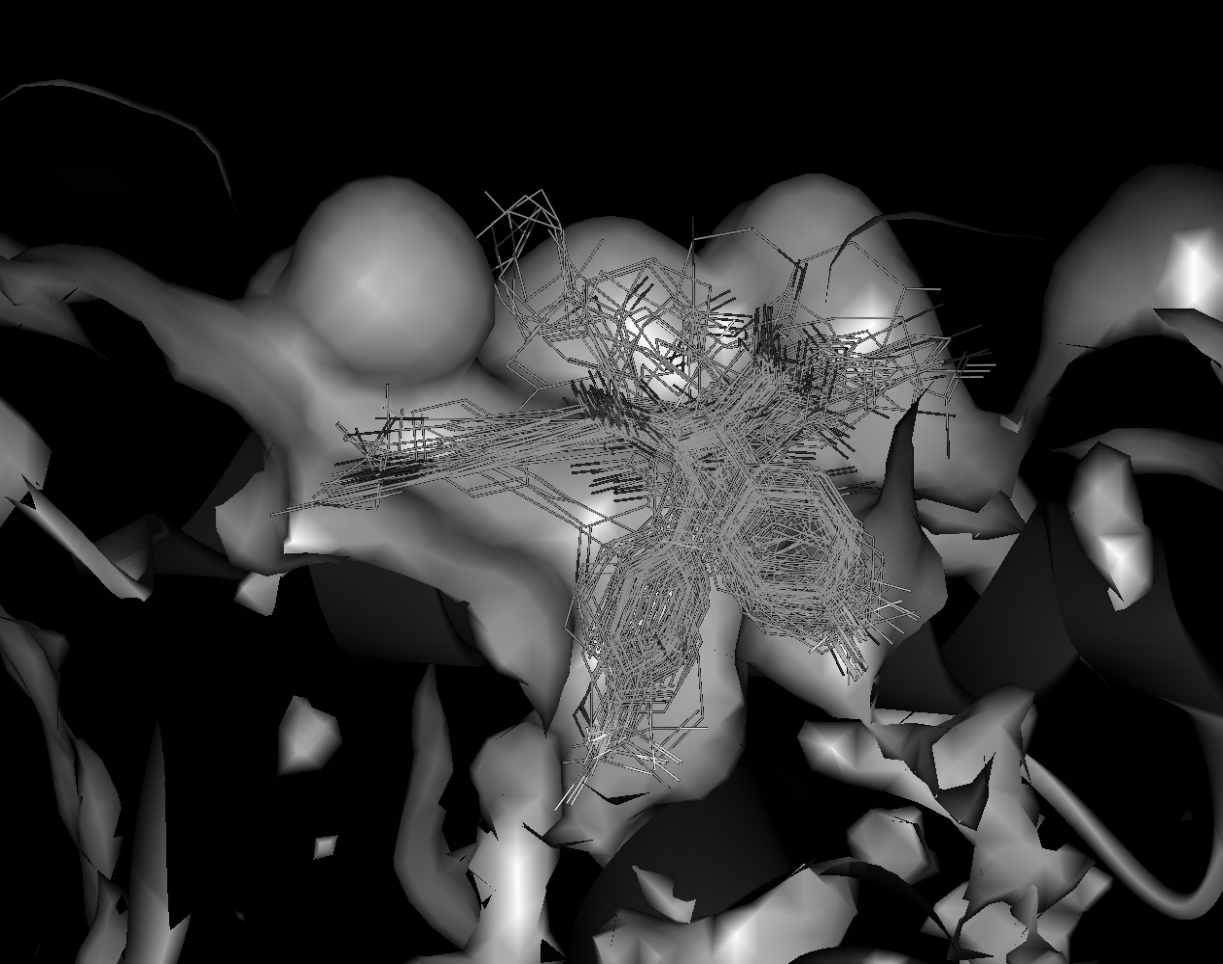
Docked positions with the minimized CDOCKER interaction energies (*E*_CD_) of the 59 1,4-benzodiazepine-2,5-diones compounds

The *FP *activity of ligands can be changed by substituted groups in aryl groups of *R*_2_ and *R*_3_. The absence of a substituent in phenyl group resulted in a dramatic loss of potency as exemplified by ligand 28. Proper substituent size in the para-position of the phenyl group will make the ligand match the HDM2 cleft more suitably and the results show that the ethyl group (ligand 10), Cl, CF_3_ and OCF_3_ (ligands 12-14) are optimal for these substituents. All three aryl groups are in the cleft of HDM2 for these ligands. Substitution at either the ortho- or meta-positions of phenyl group in *R*_2 _results in a sharp loss in activity due to the spatial clag generated for HDM2-liangd interaction (ligands 14 and 15). Figure 4D shows the docking poses comparison of ligand 12 with ligand 15. The phenyl group A in 4-10 is driven out of the cleft. To get a better understanding of the effect of 1,4-benzodiazepine-2,5-diones compounds 3D structure on their bioactivities, the docked positions with the minimized CDOCKER interaction energies (*E*_CD_) of the 59 compounds are shown in Figure 5.

Although the structural information can be obtained by viewing the ligand poses in the cleft of HDM2 and there are many SAR analyses everywhere (4, 15), these SARs cannot directly give the bioactivity values. The QSAR can give an explicit predicted value for a new compound and QSAR studies are vital to enable the prioritization of analogues resulting from iterative virtual screening and thus, the design of focused small molecule libraries around active ligands. Recently, there are some reports on QSAR study on MDM2 inhibitors (22, 42) and most recently Wang *et al. *(12) built some QSAR models using CoMFA and CoMSIA for BDPs and peptides as HDM2 inhibitors. However, there are no explicit quantitative equation model reported on 1,4-benzodiazepine-2,5-dione compounds though they are a kind of most potent entities as HMD2 inhibitors. A quantitative equation QSAR model of HMD2 inhibitors is more convenient to be used for the discovery of new antitumor drugs based on p53-HDM2 interaction. Therefore in this article, we used the protocol of Calculate Molecular Properties in DS 2.1 and E-Dragon 1.0 to calculate various physiochemical properties as the candidate descriptors for the construction of QSAR models of the substituted 1,4-benzodiazepine-2,5-dione compounds. Since the number of properties is larger than the kinds of obtained ligands, it should be reduced to a much small number than that of ligands in order to obtain an equation statistically. Due to the large number of properties, GFA method was employed to eliminate the properties with less relation to the bioactivity of ligand and keep the subset consisting of the most correlation to the potency of ligands. In this study, the physiochemical properties of BDPs from DS 2.1 were directly treated with the GFA protocol of this software, but the molecular descriptors from E-Dragon were treated with GFA in Material Studio 4.0. The linear term was used for the development of models with Friedman *LOF *smoothness parameter of 0.5 and the population size of 1000. The obtained QSAR model using linear term was then further treated with G/PLS and the model on the descriptors from DS 2.1 is as follows: 

*pIC*_50_ *= *14.568 + 0.388 LogD - 0.166 *Num_ RotatableBonds *- 0.670 *Num_StereoAtoms *+ 0.00278 *V_DIST_equ *- 1.446 *CHI_1 *- 0.0471 *Dipole_X *+ 0.230 *Shadow_Xlength *- 0.0328 *Shadow_XZ *(Equation 1) 

The sample number N = 59, *LOF *= 0.198, *R*^2^ = 0.750, R ^2^_adj=_ 0.672 = R ^2^_cv_ ,0.712 = R ^2^_adj_, *F *= 19.54. The standardized regression coefficient for each variable is 0.624, - 0.450, - 0.477, 4.01, - 3.30, - 0.546, 0.492 and -0.394 respectively. 

In our study, *R*^2^, R ^2^_cv_ , R ^2^_adj_ and *F *were used to evaluate the regression model. Equation 1 can explain 71.2% of the variance (R^ 2^_ cv_ ) while it could predict 67.2% of the variance (R^ 2^_cv_ ). *F *> *F*_(a = 0.05)_ = 2.13 shows that the model is in the confidence interval of 95%. It can be seen from Equation 1 that *LogD, V_DIST_equ *and *Shadow_Xlength *have positive contribution to the bioactivity of the ligands, however, *Num_ RotatableBonds*, *Num_StereoAtoms*, *Dipole_X*, *CHI_1*, and *Shadow_XZ *have negative effect on the bioactivities of the ligands. The relative importance of the descriptors is in the following order according to their standardized regression coefficients: 

*V_DIST_equ > CHI_1 >> LogD > Dipole_X > Shadow_Xlength > Num_StereoAtoms > Num_RotatableBonds > Shadow_XZ*

From this order, we can see that *V_DIST_equ *and *CHI_1 *play the key role in determining the bioactivity of ligands, however, since *CHI_1 *and *Shadow_XZ *have roughly the same change tendency as *V_DIST_equ*, their effect on the bioactivities of ligands is mainly counteracted by *V_DIST_equ. *Although ligands 26, 27, 56 and 57 have comparatively high values of *CHI_1 *and *Shadow_XZ, *they possess significant inhibitory activity due to the high *V_DIST_equ *values. Ligands 23, 26-29 with *R*_1_ substituents have the high *LogD *and the ligands 52, 54 and 55 with the higher *Shadow_Xlength *also have higher *pIC*_50_ values. *Num_StereoAtoms *reflects that the fewer chiral atoms a ligand has, the higher the *pIC*_50_ value it possesses (for example, ligand 1). The observed and predicted *pIC*_50_ results and the values of physiochemical properties of the 59 ligands are listed in Table 2. 

**Table 2:** Observed and predicted HDM2 inhibitory activities, physiochemical properties of different ligands from DS 2.1 used for the construction of QSAR models

**Ligand No** ***.***	***LogD***	***Num_RotatableBonds***	***Num_StereoAtoms***	***V_DIST_equ***	***CHI_1***	***Dipole_X***	***Shadow_Xlength***	***Shadow_XZ***	***pIC***_50 _ ***(Obs*** ^a^ ***)***	***pIC***_50_ ***(pred***^a^***)***	***Residual***
1	5.968	8	1	4724.72	17.24	2.226	16.104	112.169	3.071	2.995	0.076
2	6.346	8	2	4975.41	17.668	-0.232	17.246	106.927	3.155	3.099	0.056
3	6.624	9	3	5948.66	18.879	8.911	17.044	105.934	3.009	2.878	0.131
4	7.801	10	3	6357	19.352	17.426	17.251	102.209	2.983	3.388	-0.404
5	6.869	8	2	5267.12	18.206	4.827	16.575	100.892	3.149	3.140	0.009
6	6.819	9	2	5586.73	18.706	0.525	16.594	112.033	2.955	2.960	-0.005
7	4.35	5	3	4029.76	16.074	-9.636	14.335	88.627	2.658	2.206	0.452
8	2.993	4	2	2898.37	14.469	-6.569	13.227	84.085	1.42	1.444	-0.024
9	3.367	4	2	3165.49	14.863	-9.403	13.429	87.255	1.876	1.837	0.039
10	3.823	5	2	3466.24	15.401	-12.116	13.102	86.975	2.125	1.967	0.158
11	4.602	5	3	3750.51	15.774	-11.525	13.018	88.293	1.745	1.759	-0.014
12	3.544	4	2	3165.49	14.863	-4.633	15.806	86.259	2.602	2.260	0.342
13	4.929	6	3	4399.17	16.548	-4.988	15.714	85.661	2.879	2.800	0.079
14	3.935	5	3	3784.06	16.091	-3.582	14.318	84.767	0.903	1.175	-0.272
15	3.618	5	3	3903.07	16.074	-10.055	13.477	89.093	1.347	1.377	-0.030
16	3.866	4	3	3165.49	14.863	-7.035	14.801	88.055	1.194	1.538	-0.344
17	4	7	2	3034.57	14.329	-5.412	17.163	98.372	1.854	2.298	-0.444
18	4.251	7	3	3290.34	14.684	-2.954	18.145	106.53	1.921	1.765	0.156
19	3.909	5	2	3473.35	15.346	-2.056	13.726	83.834	1.921	1.872	0.049
20	4.659	4	2	3442.8	15.257	-11.197	14.262	90.403	2.799	2.712	0.087
21	5.371	5	3	4050.07	16.168	-3.294	17.258	99.523	2.81	2.538	0.272
22	5.191	5	3	4342.96	16.468	-3.431	18.061	100.242	2.644	3.015	-0.371
23	5.116	5	3	4342.96	16.468	-9.904	15.338	90.364	3.174	2.989	0.185
24	3.117	4	2	3442.8	15.257	-3.545	16.27	87.575	1.783	2.306	-0.523
25	4.793	4	2	3442.8	15.257	-7.81	15.663	92.037	3.377	2.872	0.505
26	4.333	4	2	3442.8	15.257	-5.83	17.149	91.669	3.208	2.954	0.254
27	5.071	5	3	4329.62	16.468	-8.796	16.683	97.357	3.208	2.962	0.246
28	5.593	6	3	4709.4	16.941	-7.532	17.39	98.783	3.119	3.425	-0.306
29	4.316	4	2	3402.78	15.257	-7.623	14.344	90.543	2.569	2.313	0.256
30	4.222	4	2	3377.34	15.274	-2.818	14.732	85.308	1.699	2.216	-0.517
31	3.531	4	2	3204.98	14.863	11.824	17.042	94.786	1.83	1.594	0.236
32	4.869	5	2	3713.52	15.795	-1.206	16.846	93.799	2.824	2.612	0.212
33	5.121	5	3	3982.18	16.168	-9.203	16.697	96.658	2.991	2.495	0.496
34	5.327	5	3	4252.1	16.468	-8.908	16.416	95.685	2.644	2.845	-0.201
35	4.919	4	2	3442.8	15.257	-8.057	16.867	94.102	2.815	3.142	-0.327
36	4.419	4	2	3442.8	15.257	-5.742	16.661	89.394	3.013	2.946	0.067
37	4.53	4	2	3419.09	15.274	-7.345	15.007	89.47	2.201	2.592	-0.391
38	5.109	4	2	3662.15	15.684	-8.063	14.67	91.643	3.155	2.784	0.371
39	3.322	5	2	3713.52	15.795	-11.792	16.856	94.882	2.481	2.477	0.004
40	3.643	6	2	4021.16	16.295	-12.178	16.612	96.058	2.62	2.490	0.130
41	3.127	5	2	4319.03	16.651	20.781	15.821	96.509	0.903	1.020	-0.117
42	4.171	4	2	3713.52	15.795	-7.892	16.853	93.853	1.83	2.822	-0.992
43	5.008	4	2	3713.52	15.795	-4.955	16.946	95.986	3.06	2.960	0.100
44	2.627	4	2	3442.8	15.257	-7.775	15.555	90.193	2.833	2.065	0.768
45	3.847	4	2	3442.8	15.257	-7.717	15.455	90.231	2.131	2.512	-0.381
46	3.901	4	2	3897.87	16.346	16.632	17.378	98.099	1.26	1.259	0.001
47	4.649	4	2	3944.98	16.329	-9.054	15.086	94.999	2.102	2.490	-0.388
48	6.346	8	2	4975.41	17.668	12.051	15.952	92.366	3.068	2.701	0.367
49	4.11	6	2	5227	18.329	-0.345	15.151	94.6	2.569	2.234	0.335
50	4.11	6	2	5227	18.329	0.53	15.821	90.138	2.204	2.493	-0.289
51	4.382	6	2	5512.31	18.74	-0.688	18.153	106.981	3.435	2.837	0.598
52	4.382	6	2	5512.31	18.74	-0.869	15.882	107.351	1.883	2.312	-0.429
53	5.581	9	2	5015.05	17.812	-0.683	17.066	100.792	2.622	2.718	-0.096
54	5.581	9	2	5015.05	17.812	-1.101	17.187	113.343	1.903	2.354	-0.451
55	4.803	9	2	5294.3	18.222	-0.456	19.139	102.328	3.405	3.013	0.392
56	5.404	7	2	6151.45	19.778	0.656	18.773	117.298	3.104	3.082	0.022
57	3.694	7	2	6364.86	19.634	-0.715	16.251	99.847	3.263	3.276	-0.013
58	2.553	5	2	5791.5	19.151	-0.666	17.56	107.372	2.268	2.323	-0.055
59	3.913	5	2	5791.5	19.151	-0.321	15.988	93.512	2.81	2.929	-0.119

aObs, observed. bPred, predicated

**Figure 6:**
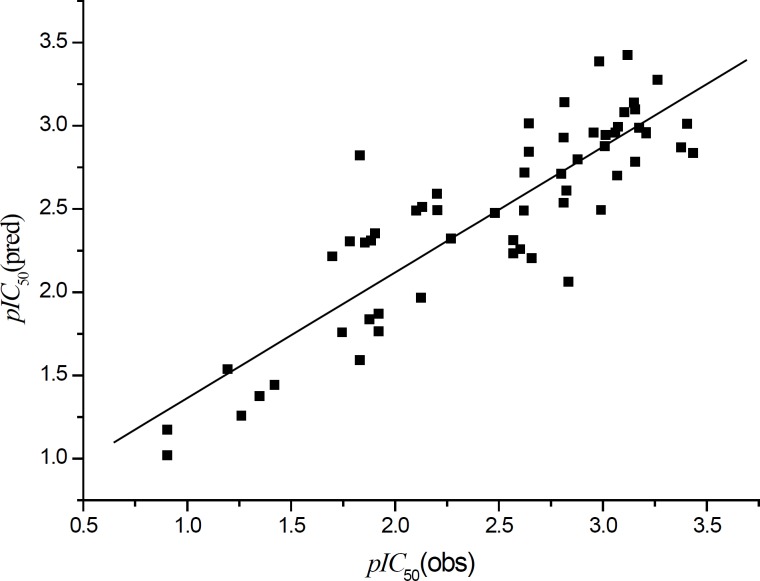
Plot of observed vs. predicted HDM2 inhibitory activities of different ligands in Table 1 with Equation 1

The plot of the observed *pIC*_50_ vs. the predicted data is shown in Figure 6.

 It can be seen that the predicted data by this model is basically in accordance with the experimental results. As a whole, it is only considered as a moderate QSAR model. In order to further improve the model quality, obtaining more descriptors is necessary. Thus, we collected 1620 kinds of molecular descriptors of BDPs using E-Dragon online tool. The QSAR model was obtained using GFA in MS 4.0. The QSAR model obtained is as follows: 

*pIC*_50_ = 7.858 *IDE *- 4.855 *MATS7v *- 1.198 DP09 - 0.448 *Mor14m *+ 1.481 *Mor30p *- 25.917 *G2e *+ 1.678 *E2e *+ 0.319 Tp + 61.575 R5u - 61.64 *BELp6 *+ 0.272 *SeaC2C3aa *- 12.276 

(Equation 2) 

The sample number *N *= 59, *LOF *= 0.161, *R*^2^ = 0.882, 0.817 = R ^2^_cv_ ,0.855 = R ^2^_ adj_ , F = 31.98 

The observed and predicted *pIC*_50_ results and the values of physiochemical properties of the 59 ligands for Equation 2 are listed in Table 3. 

**Table 3:** Observed and predicted HDM2 inhibitory activities, physiochemical properties of different ligands from E-Dragon 1.0 used for the construction of QSAR models

**Ligand No.**	***IDE***	***MATS7v***	***DP09***	***Mor14m***	***Mor30p***	***G2e***	***E2e***	***Tp***	***R5u***	***BELp6***	***SeaC2C3aa***	***pIC***_50_ **(Obs** ^a^ **)**	***pIC***_50_ **(pred** ^a^ **)**	**Residual**
1	3.551	-0.107	9.455	1.209	-0.032	0.165	0.415	18.765	0.031	1.478	12	3.071	2.511	0.560
2	3.64	0.027	10.342	0.029	0.028	0.142	0.45	21.391	0.025	1.478	12	3.155	3.236	-0.081
3	3.707	0.017	11.604	-0.689	-0.117	0.149	0.404	22.48	0.03	1.478	12	3.009	2.803	0.206
4	3.566	-0.059	10.281	-0.078	0.154	0.15	0.282	20.91	0.032	1.515	12	2.983	2.936	0.047
5	3.593	-0.09	10.593	0.033	0.154	0.167	0.377	22.84	0.033	1.555	12	3.149	3.019	0.130
6	3.394	-0.027	8.432	-0.047	-0.133	0.151	0.517	15.905	0.027	1.248	10	2.955	2.806	0.150
7	3.394	-0.027	9.424	-0.994	-0.102	0.151	0.647	17.59	0.027	1.248	10	2.658	2.842	-0.184
8	3.113	-0.156	7.799	0.2	-0.012	0.163	0.464	15.146	0.029	1.223	8	1.42	1.145	0.275
9	3.191	-0.162	7.992	0.486	-0.154	0.16	0.506	15.639	0.026	1.232	10	1.876	1.826	0.050
10	3.287	-0.166	8.95	0.064	0.015	0.166	0.61	18.711	0.032	1.389	10	2.125	2.271	-0.146
11	3.35	-0.17	9.216	0.823	-0.142	0.147	0.49	19.924	0.027	1.412	10	1.745	2.119	-0.374
12	3.191	-0.162	8.596	-0.068	0.118	0.173	0.662	16.791	0.029	1.223	10	2.602	2.287	0.315
13	3.504	-0.043	8.75	-1.098	0.031	0.168	0.432	15.764	0.029	1.357	10	2.879	2.890	-0.011
14	3.175	-0.062	8.281	-0.203	0.015	0.156	0.457	16.098	0.024	1.301	8	0.903	0.493	0.410
15	3.281	0.071	8.697	-0.369	-0.157	0.151	0.496	17.12	0.03	1.276	10	1.347	1.594	-0.247
16	3.191	-0.162	8.611	0.469	0.013	0.158	0.369	17.544	0.027	1.236	8	1.194	1.260	-0.066
17	3.27	-0.188	8.866	0.735	0.047	0.167	0.37	19.087	0.037	1.298	8	1.854	2.118	-0.264
18	3.326	-0.197	8.812	0.842	0.13	0.147	0.363	17.767	0.025	1.325	8	1.921	1.919	0.002
19	3.294	-0.181	9.724	-0.902	-0.231	0.188	0.567	19.389	0.029	1.224	10	1.921	1.967	-0.046
20	3.263	-0.172	9.12	0.405	0.109	0.169	0.323	19.086	0.026	1.226	12	2.799	2.655	0.144
21	3.412	-0.178	9.676	0.648	0.074	0.175	0.332	21.844	0.024	1.402	12	2.81	2.536	0.274
22	3.457	-0.181	8.038	0.767	-0.041	0.161	0.355	16.094	0.024	1.409	12	2.644	3.168	-0.524
23	3.457	-0.042	9.973	-0.727	-0.051	0.151	0.467	17.533	0.027	1.237	12	3.174	3.002	0.172
24	3.263	-0.041	9.113	0.254	0.258	0.172	0.352	18.119	0.026	1.223	12	1.783	1.997	-0.214
25	3.263	-0.172	8.089	-0.436	0.07	0.163	0.326	15.73	0.023	1.223	12	3.377	3.134	0.243
26	3.263	-0.249	9.22	-0.433	-0.088	0.163	0.413	18.672	0.024	1.223	12	3.208	3.063	0.145
27	3.446	-0.042	9.62	-0.84	0.082	0.16	0.35	18.476	0.026	1.248	12	3.208	3.326	-0.118
28	3.549	-0.059	9.634	0.031	-0.071	0.16	0.52	17.239	0.031	1.357	12	3.119	3.098	0.021
29	3.223	-0.162	8.958	-0.578	0.207	0.153	0.333	17.8	0.025	1.223	12	2.569	3.050	-0.481
30	3.197	-0.16	7.767	-0.256	0.096	0.183	0.351	14.796	0.026	1.223	10	1.699	1.767	-0.068
31	3.234	-0.185	8.014	-0.299	0.068	0.173	0.46	14.406	0.028	1.253	10	1.83	2.113	-0.283
32	3.315	-0.172	8.436	-0.219	0.1	0.158	0.301	16.152	0.028	1.401	12	2.824	2.484	0.340
33	3.352	-0.159	8.837	-0.078	0.192	0.156	0.317	17.405	0.029	1.412	12	2.991	2.775	0.216
34	3.381	-0.148	9.993	-0.42	0.122	0.157	0.365	21.164	0.026	1.416	12	2.644	2.657	-0.013
35	3.263	-0.186	9.034	-0.838	0.194	0.153	0.302	16.222	0.023	1.236	12	2.815	2.727	0.088
36	3.263	-0.192	8.902	-1.076	0.013	0.163	0.498	15.55	0.029	1.231	12	3.013	3.010	0.003
37	3.24	-0.195	8.036	-0.692	0.035	0.168	0.372	14.139	0.031	1.252	10	2.201	2.398	-0.197
38	3.266	-0.172	8.297	0.308	0.16	0.158	0.356	15.762	0.032	1.223	10	3.155	2.908	0.247
39	3.315	-0.2	8.874	-0.679	0.098	0.15	0.332	15.685	0.03	1.367	12	2.481	2.746	-0.265
40	3.387	0.022	8.081	-0.806	0.027	0.148	0.509	15.319	0.022	1.41	12	2.62	2.605	0.015
41	3.437	0.002	9.481	-0.689	0.197	0.196	0.31	17.995	0.028	1.423	12	0.903	1.181	-0.278
42	3.315	-0.2	9.572	-0.557	0.218	0.172	0.392	16.828	0.024	1.316	12	1.83	1.880	-0.050
43	3.315	-0.172	9.301	-1.091	0.231	0.166	0.342	17.812	0.03	1.337	12	3.06	2.949	0.111
44	3.263	-0.121	8.121	0.392	0.256	0.174	0.34	15.488	0.033	1.223	11	2.833	2.758	0.075
45	3.263	-0.102	9.158	0.138	-0.057	0.153	0.399	18.089	0.025	1.223	11	2.131	2.052	0.079
46	3.277	-0.106	8.706	-0.81	-0.062	0.16	0.416	16.839	0.022	1.406	11	1.26	1.254	0.006
47	3.319	-0.187	8.964	-0.322	-0.14	0.177	0.538	17.723	0.026	1.367	12	2.102	2.144	-0.042
48	3.551	-0.107	10.267	-0.359	0.172	0.155	0.506	20.493	0.026	1.478	12	3.068	3.197	-0.129
49	3.537	-0.083	9.321	1.053	0.073	0.142	0.413	18.866	0.025	1.497	12	2.569	2.806	-0.237
50	3.537	-0.083	10.834	0.97	0.222	0.162	0.408	23.122	0.029	1.497	12	2.204	2.327	-0.123
51	3.543	-0.033	10.826	-0.034	0.286	0.148	0.405	23.239	0.029	1.499	12	3.435	3.068	0.367
52	3.543	-0.033	11.07	0.938	0.449	0.166	0.378	23.406	0.026	1.499	12	1.883	1.939	-0.056
53	3.581	-0.061	10.664	0.938	0.257	0.163	0.416	23.293	0.027	1.511	12	2.622	2.667	-0.045
54	3.581	-0.061	10.691	0.493	0.024	0.143	0.394	22.118	0.02	1.511	12	1.903	2.164	-0.261
55	3.585	-0.018	9.081	-0.211	0.084	0.149	0.457	18.393	0.02	1.512	12	3.405	3.077	0.328
56	3.581	0.001	9.405	-0.376	0.145	0.139	0.501	19.113	0.018	1.571	12	3.104	2.798	0.306
57	3.711	-0.019	8.968	0.722	0.136	0.15	0.444	18.373	0.019	1.578	12	3.263	3.336	-0.073
58	3.533	-0.035	10.559	-0.253	0.223	0.156	0.37	21.596	0.025	1.494	12	2.268	2.319	-0.051
59	3.54	0.002	9.656	-0.41	0.218	0.141	0.489	19.593	0.025	1.507	12	2.81	3.208	-0.398
I	3.577	-0.133	9.438	0.207	0.253	0.154	0.46	19.523	0.022	1.617	14		3.451	
II	3.565	-0.06	9.274	0.328	0.323	0.147	0.414	18.497	0.022	1.617	14		3.025	

Equation 2 can explain 85.5% of the variance (*R*^2^_cv_ ) while it could predict 81.7% of the variance (*R*^2^_cv_ ). *F *> *F*_(a = 0.05)_ = 2.13 shows that the model is in the confidence interval of 95%. This model shows *IDE, Mor30p, E2e, Tp, R5u *and *SeaC2C3aa*. *Count16 *give positive contribution to the bioactivity of the ligands but *MATS7v*, *Mor14m, G2e *and *BELp6 *have the negative effect on the bioactivities of BDPs. The standardized regression coefficient for each variable is 1.772, -0.559, - 1.621, - 0.416, 0.300, -0.453, 0.217, 1.192, 0.336, -1.106 and 0.520 respectively. The relative importance of the descriptors according to their standardized regression coefficients is in the following order: 

*IDE > DP09 > Tp > BELp6 > MATS7v > SeaC2C3aa.Count16 > G2e > Mor14m > R5u > Mor30p > E2e*

**Figure 7:**
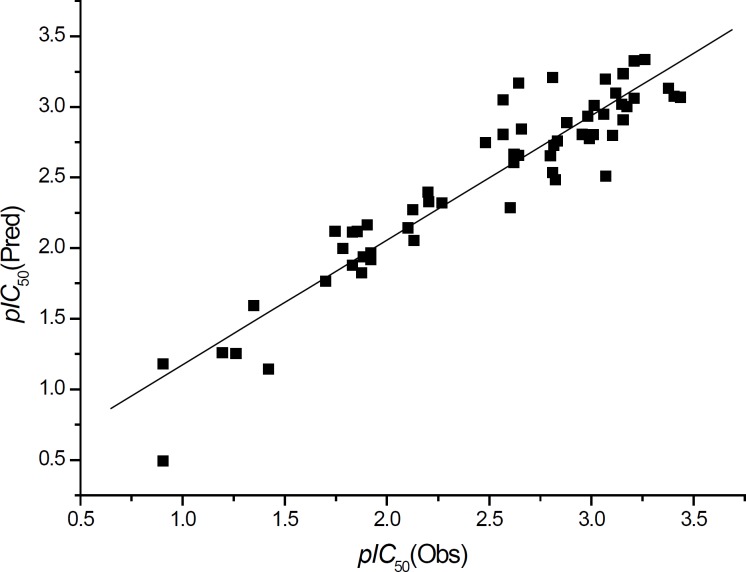
The plot of the observed *pIC*_50_ vs. the predicted data

The plot of the observed *pIC*_50_ vs. the predicted data with Equation 2 is shown in Figure 7 and it illustrates that Equation 2 is a better model compared with Equation 1. 

*Prediction of some new HDM2 inhibitors*

**Figure 8:**
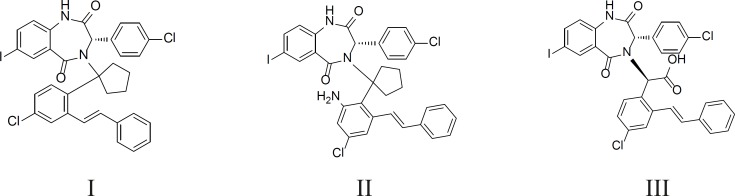
Some new compounds designed by us

**Figure 9:**
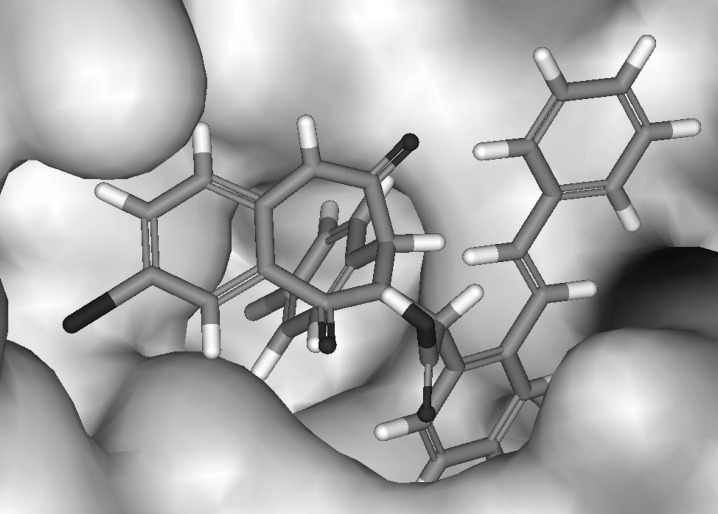
Docked pose of compound III

Based on the mentioned analysis, we designed some new compounds (Figure 8, I-III) and evaluated them with Equation 2. The predicted bioactivities are listed in Table 3. These compounds have higher inhibitory potency and their activities can be verified through chemosynthesis and *FP *assay lately. The docked pose of III using CDOCKER is shown in Figure 9. 

From the structure of these coumpounds, we can see that the cyclopentane group gives the stability of the ligand conformation accormadating the cleft space. The extra aryl group at *R*_3_ improved the hydrophobic interaction between HDM2 and the ligands. It is to be clarified that this model is based on the molecule-based (*FP *assay) potency. Considering the cellular activity of these compounds, the water-solubility should be improved through introducing some polar groups. From this point, compound III may be more applicable as potent HDM2 inhibitors.

## Conclusions

In order to explore appropriate binding mode of different 1,4-benzodiazepine-2,5-dione compounds as HDM2 inhibitors, CDOCKER protocol in DS 2.1 was employed to conduct the docking process using a dataset of 59 1,4-benzodiazepine-2,5-dione compounds from literatures (4, 15, 31, 32) and the binding situation was analyzed. Our docking pose of freshly prepared model of ligand 25 corroborates to the crystal structure, which indicates the reliability of this docking procedure. The docking results indicate that hydrophobic interaction between the imidazole group in HIS96 and the aryl ring in *R*_3_ may be one of the key factors for the combination of the ligands with HDM2. The binding cleft of HDM2 is predominantly hydrophobic and largely nonspecific Van Der Waals contacts are responsible for the interaction between the ligand and HDM2 hydrophobic pocket. Although the pocket of HDM2 is mainly hydrophobic, the hydrogen bonds can be formed with VAL93 residue in HDM2 for some ligands. The extra hydrogen bond formation can increase the inhibitory potency of the ligands to some extent. The chirality species of substituents in *R*_1_ to *R*_4_ influence the ligands’ potency seriously. Various 1D-3D physiochemical properties such as structural and Jurs parameters, and also topological descriptors from DS2.1 and E-Dragon 1.0 were selected as candidate descriptors to build two QSAR models separately. The QSAR model Equation 1 (with eight descriptors) built using more than 100 descriptors from DS2.1 was obtained. It can explain 71.2% of the variance (*R*^2^_adj_ ) while it could predict 67.2% of the variance (*R*^2^_adj_ ). *F *> *F*_(a = 0.05)_ = 2.13 shows that the model is in the confidence interval of 95%. In order to improve the QSAR model quality, more than 1600 descriptors obtained from E-Dragon 1.0 were used to build the QSAR model with 11 descriptors (Equation 2). It can explain 85.5% of the variance (*R*^2^_adj_ ) while it could predict 81.7% of the variance (*R*^2^_cv_). For both models, *F *> *F*_(a = 0.05)_ = 2.13 shows that they are in the confidence interval of 95%. With these models, the bioactivities of some new compounds were predicted. The results show that the formation of cyclopentane ring at *α*-C or introduction of extra aryl group at original phenyl group of R_3_ can improve the ligands’ predicted *pIC*_50_ values, which may improve the HDM2 inhibitory potency.
